# JNK inhibitor alleviates apoptosis of fetal neural stem cells induced by emulsified isoflurane

**DOI:** 10.18632/oncotarget.21505

**Published:** 2017-10-04

**Authors:** Lei Zhou, Zeyong Yang, Xianfu Lu, Xingxing Li, Xiaohu An, Jing Chai, Qiling Yang, Shikai Yan, Yuanhai Li

**Affiliations:** ^1^ Department of Anesthesiology, First Affiliated Hospital of AnHui Medical University, Hefei 230022, PR China; ^2^ Department of Anesthesiology, International Peace Maternity and Child Health Hospital, Shanghai Jiao Tong University School of Medicine, Shanghai 200030, PR China; ^3^ School of Pharmacy, Shanghai Jiao Tong University, Shanghai 200240, PR China

**Keywords:** fetal neural stem cells, emulsified isoflurane, apoptosis, JNK, neurotoxicity

## Abstract

Isoflurane can provide both neuroprotection and neurotoxicity in various culture models and in rodent developing brains. Emulsified Isoflurane (EI) is an emulsion formulation of isoflurane, while its underlying molecular mechanism of developemental nerve toxicity largely remains unclear. We hypothesized that EI induced fetal neural stem cells (FNSCs) apoptosis, endoplasmic reticulum (ER) stress and c-Jun N-terminal kinase (JNK) activation.

FNSCs were isolated from the cortex of SD rats during 14 days of gestation. The cell viability, cell apoptotic rates and the expression of apoptosis-related protein Caspase3, inositol requiring enzyme 1 (IRE1), poly (adenosine diphosphate-ribose) polymerase (PARP), Bax, Bcl-2, JNK, p-JNK and XBP1 were determined. Specific inhibition was performed by siRNA-targeting of JNK in FNSCs. EI could increase the p-JNK, JNK and caspase3 protein expression, the JNK pathway was activated by EI, and EI-induced apoptosis was blocked by inhibiting JNK pathway with SP600125 or JNK-small interfering RNA (siRNA), EI enhanced the level of IRE1, PARP, Bax/Bcl-2 and XBP1, which led FNSCs to apoptosis and ER stress. Meanwhile, dilatation of the ER lumens in FNSCs treated by EI for 24 h was significant. G*reen* fluorescent protein (GFP) positive cell ratios were significantly decreased by FNSCs transfecting with JNK gene silencing. JNK was efficiently silenced in siRNA-JNK1 group.

The results provided *in-vitro* evidence which supports that the underlying mechanisms of EI-induced apoptosis are the induction of ER stress and sequent JNK activation. Together, these data suggest that JNK inhibiting might be applied for improving therapeutic outcomes in anesthestics-induced neurotoxicity.

Highlights:

1. Prolonged treatment with high-dose EI decreased the survival level of FNSCs by inducing apoptosis and inhibiting proliferation via the JNK signaling pathway.

2. EI induced ER stress and sequent JNK activation.

3. JNK inhibiting might be applied for improving therapeutic outcomes in anesthestics-induced neurotoxicity

## INTRODUCTION

Anesthetic agents induce cell apoptosis, cause synaptic remodeling, and alter the morphology of the developing nerve [1–5]. Moreover, children exposed to anesthesia in early life have a higher incidence of learning deficits in adolescence [6]. Endoplasmic reticulum (ER) is a key organelle to participate in a variety of cell processes, such as maintaining caspase activation, which play an important role in the maintenance of cell survival and the normal physiological function of cells.

Conditions that disrupt the ER function cause an accumulation of misfolded proteins, whose turnover is usually mediated by ER-associated degradation [7, 8], which alleviates this stress and restores ER homeostasis, increasing cell viability and adaptation [9, 10]. There are some evidences showing that isoflurane could cause ER stress (inducing caspase-12 activation) in primary neurones [11]. JNK signaling exists in the ER stress response at early prosurvival phase. Activated JNK can be triggered by Aβ-induced toxicity on neuronal apoptosis and long-term potentiation deficit *in vitro* [12, 13], and ER stress and memory impairment *in vivo* [14, 15]. Previous studies indicate that activated JNK plays a key role in tau hyperphosphorylation and the development of amyloid plaques *in vitro* and *in vivo* [16–20].

Isoflurane have a certain cellular cytotoxicity to human neural precursor cells which can influence development of nervous system [21], and Emulsified Isoflurane (EI) is an emulsion formulation of isoflurane. however, how EI goes in developmental neural cell apoptosis when JNK activation blocked? Its underlying molecular mechanism largely remains unclear until now.

We hypothesized that that EI induced fetal neural stem cells (FNSCs) apoptosis, endoplasmic reticulum (ER) stress and JNK activation, and JNK blocker would potentially contribute to alleviate neurotoxicity induced by EI. In this study, FNSCs neurotoxicity induced by EI was investigated with SP600125 used as a small-molecule JNK-specific inhibitor. This work is expected to reveal the underlying molecular mechanism of cellular toxicity by EI, and may provide theoretical basis for the development of approaches clinically improving therapeutic outcomes in anesthestics-induced neurotoxicity.

## RESULTS

### Immunofluorescence assay of FNSCs

**Figure 1:**
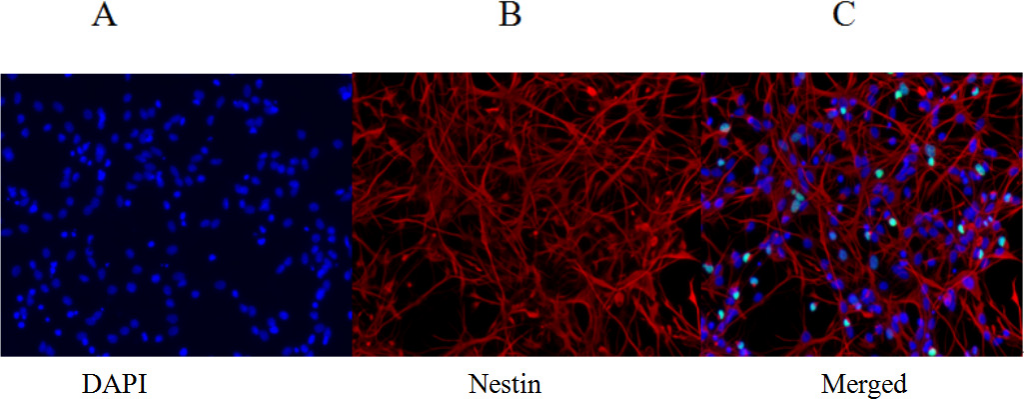
Identification of fourth-passage FNSCs after 1 day in culture (immunofluorescence staining) **(A)** DAPI nuclear staining (blue fluorescence). **(B)** Nestin-marked FNSCs (red fluorescence). **(C)** Merged image from DAPI nuclear staining and nestin-marked FNSCs. DAPI: 4′, 6-diamidino-2-phenylindole. Scale (magnifications: 200×).

The fourth passage of FNSCs was digested with 0.25% trypsin, and the digestion was terminated with culture medium containing the serum. The cell suspension was dropped onto the cover slips for 1 days of incubation. Nestin immunocytochemistry was used to identify the cultured cells as FNSCs. Red-staining nestin-immunoreactivity showed that the red color mainly existed in the cytoplasm. 4’, 6-Diamidino-2-phenylindole (DAPI) was used to label nuclei, which appeared blue. Together, this confirmed that the cultured cells were FNSCs (Figure 1A-1C).

### EI induced apoptosis of FNSCs with IRE1- JNK pathway involved

**Figure 2:**
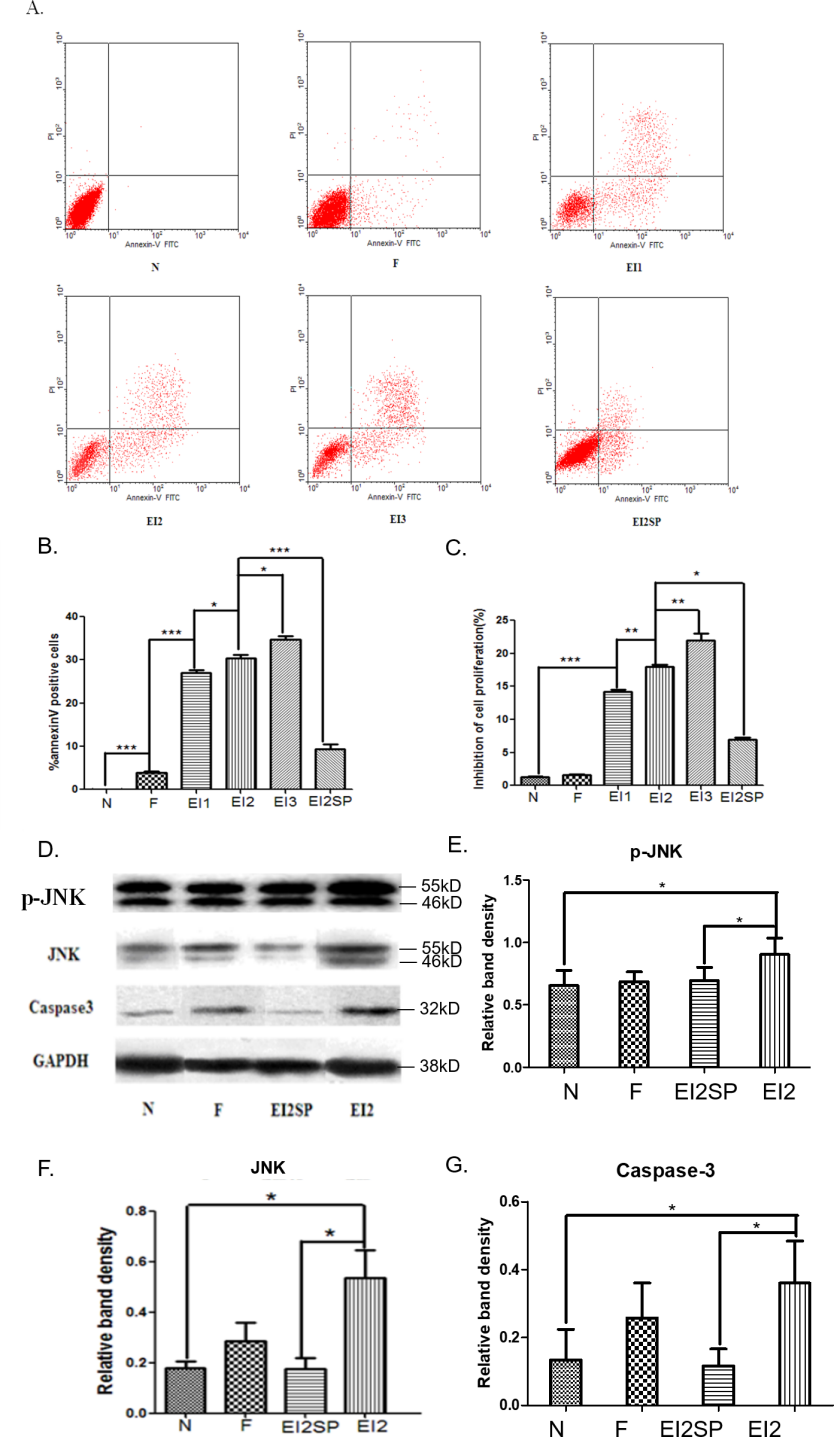
**(A)** Cells were treated with different concentrations of EI and cultured for 12hr, then analyzed by flow cytometry. **(B)** Annexin V-FITC and PI staining after EI treatment. Cells were treated with EI for 12hr. Annexin positive cell ratio of FNSCs after EI treatment and JNK blocker SP600125. **(C)** Inhibitory ratio of cell proliferation in all groups. **(D)** The level of p-JNK, JNK and Caspase3 was determined by immunoblot analysis. GAPDH was used as a loading control. **(E, F)** JNK inhibition by the specific inhibitor SP600125 rescued FNSCs from a decrease in survival and upregulated cell viability. JNK-dependent apoptosis was inhibited by JNK blocker SP600125. The expression of p-JNK and JNK protein in FNSCs was increased by EI in comparison with N group. **(G)** Caspase3 was activated by EI with immunoblot analysis. JNK blocker SP600125 can decrease the expression of Caspase3 in group EI2SP. All data were derived form results of three independent experiments. Values are expressed as the mean ± standard deviation (SD). ^*^P<0.05, ^**^P<0.01, ^***^P<0.001. Normal group (group N), Intralipid group (group F), EI of 7.56mM (group EI1), EI of 9.52mM (group EI2), EI of 11.48mM (group EI3) and EI of 9.52mM plus SP600125 of 20μM (group EI2SP).

The result showed us that EI could inhibit the proliferation of FNSCs significantly when the cells were exposed to EI of concentration higher than 7.56mM (not shown). Cell apoptosis was significantly increased in EI-treated groups with compared with control group or intralipid-treated group (Figure 2A). The cell proliferation inhibitory rates of all EI-treated groups were higher than that of group N and F in a concentration-dependent way (Figure 2B, 2C). The expression of apoptosis-related protein caspase3, p-JNK and JNK was up-regulated by EI (Figure 2D), Even more, The effectiveness of JNK1/2 inhibition was next assessed. with SP600125 added, treatment of FNSCs exposed to EI with the JNK inhibitor SP600125 for 24 h resulted in an obvious inhibition of JNK phosphorylation, which remained lower inhibition of cell proliferation and annexin positive cells ratio in EI2SP group compared with EI2 group (Figure 2B, 2C); JNK-dependent apoptosis and caspase3 was inhibited by JNK blocker SP600125 in western blot analysis (Figure 2D). The level of p-JNK, JNK and caspase3 was determined by immunoblot analysis (Figure 2E-2G). The expression of apoptosis-related protein caspase3, p-JNK and JNK was obviously increased by EI in comparison with N group, meanwhile, the apoptosis effect was significantly decreased by JNK inhibitor SP600225 (Figure 2E-2G).

### Western blot analysis of IRE1, PARP, Bax, Bcl-2 and XBP1 proteins expression in FNSCs exposed to EI cultured for 24h

**Figure 3:**
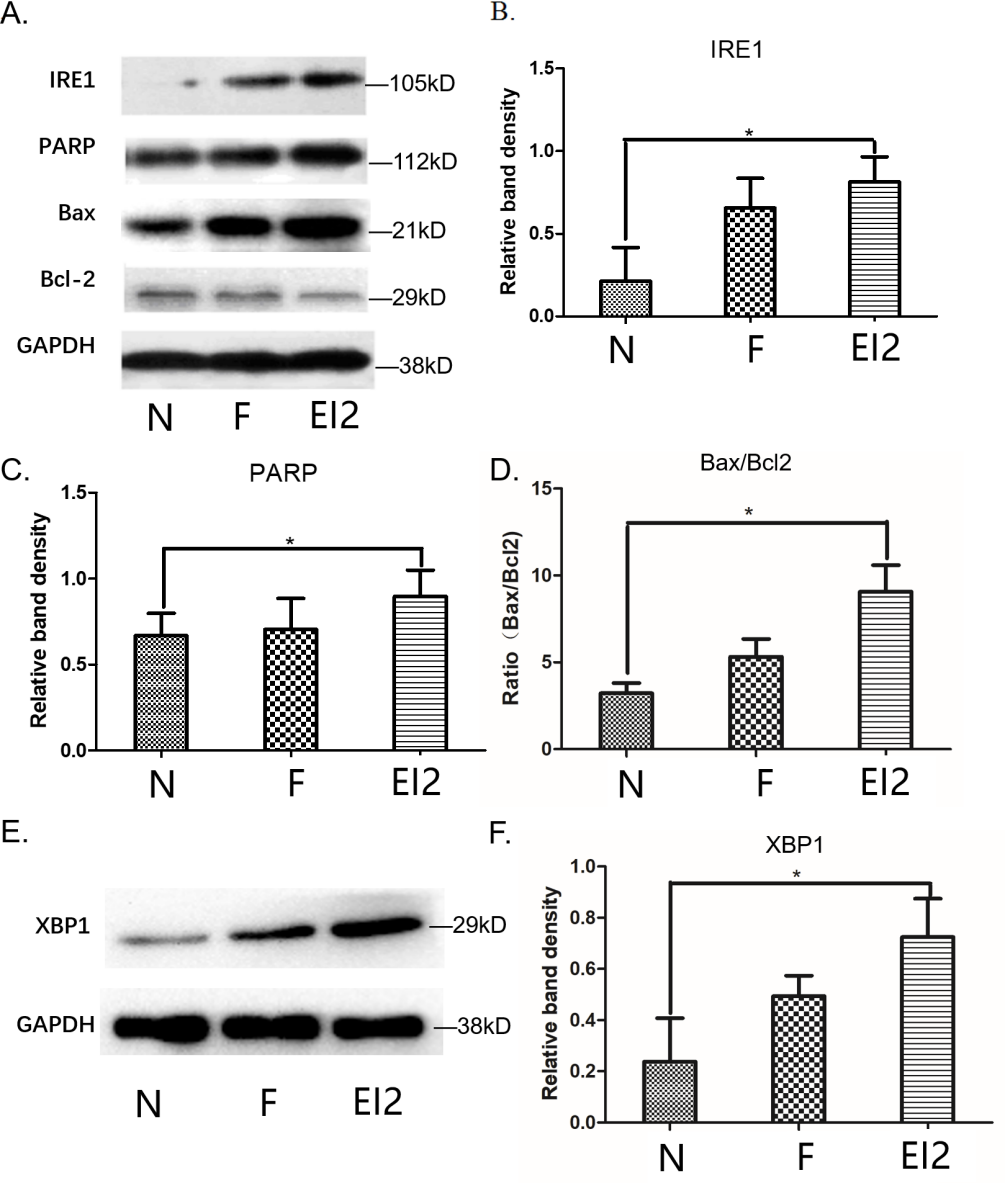
**(A)** Western blot analysis of IRE1, PARP, Bax, Bcl-2 and XBP1 proteins and cultured for 24h. **(B)** Cells were treated with 9.52mM EI and cultured for 24h, IRE1 protein expression was significantly increased. **(C)** Cells were treated with 9.52mM EI and cultured for 24h, PARP protein expression was significantly increased. **(D)** Cells were treated with 9.52mM EI and cultured for 24h, Bax/Bcl-2 ratio was significantly increased. **(E)** Western blot analysis of XBP1 proteins. **(F)** Cells were treated with 9.52mM EI and cultured for 24h, XBP1 protein expression was significantly increased. All data were derived form results of three independent experiments and are shown as mean ± SD. ^*^P<0.05 vs related group control. we analyzed by one-way ANOVA followed by Tukey multiple comparison tests. Normal group (group N), Intralipid group (group F), EI of 9.52mM (group EI2).

We performed Western blot analysis to examine the expression of IRE1 and PARP cleavage which may involve some signals indicating the appearance of apoptosis mediated by ER stress. Inositol requiring enzyme-1 (IRE1), an important ER transmembrane stress sensor. The results showed that EI exposure enhanced the activity of IRE1 and PARP, meanwhile, the expression of Bax and Bcl-2 protein was measured when FNSCs were exposed to EI for 24h (Figure 3A). According to the western blotting results, protein levels of IRE1, PARP, Bax, Bcl-2 and XBP1 were detected in FNSCs. It was reported that persistent activation of IRE1 could initiates the phosphorylation of JNK. We detected a significant increase in IRE1 protein when FNSCs were treated with EI as compared with the control group (Figure 3B). Meanwhile, we detected a significant increase in PARP and Bax/Bcl-2 protein when FNSCs were treated with EI as in comparison to the control group, whichreflected the apoptotic state of the FNSCs (Figure 3C, 3D). In the meantime, we detected an obvious rise in XBP1 protein when FNSCs were treated with EI as in comparison to the control group, whichreflected the stress state of ER (Figure 3E, 3F).

### Significant swelling of endoplasmic reticulum in FNSCs treated with EI for different durations

**Figure 4:**
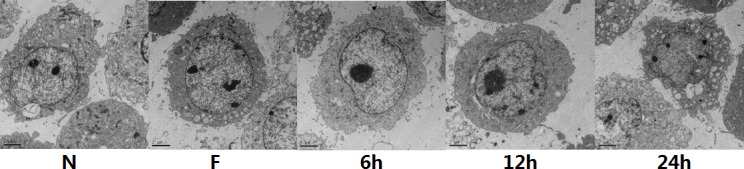
FNSCs were observed under Transmission Electron Microscope (TEM) treated with 9.56mM EI for different durations (6 h, 12 h and 24 h) Normal control cell ultrastructure with normal mitochondria and ER are shown in Control. Significant swelling of ER (in 12 h and 24 h) were observed in FNSCs of rat treated with EI. Scale bars indicate 2 μm. Normal group (group N), Intralipid group (group F).

To identify the mechanism of EI-induced apoptosis in FNSCs, we examined ultrastructural morphology of cells incubated with EI for 24h by electron microscopy. We found that dilatation of the ER lumens was significant when compared with the ER of the control group (Figure 4).

### JNK was efficiently activated in group EI2NJ (negative-siRNA+EI2) analyzed by flow cytometry

**Figure 5:**
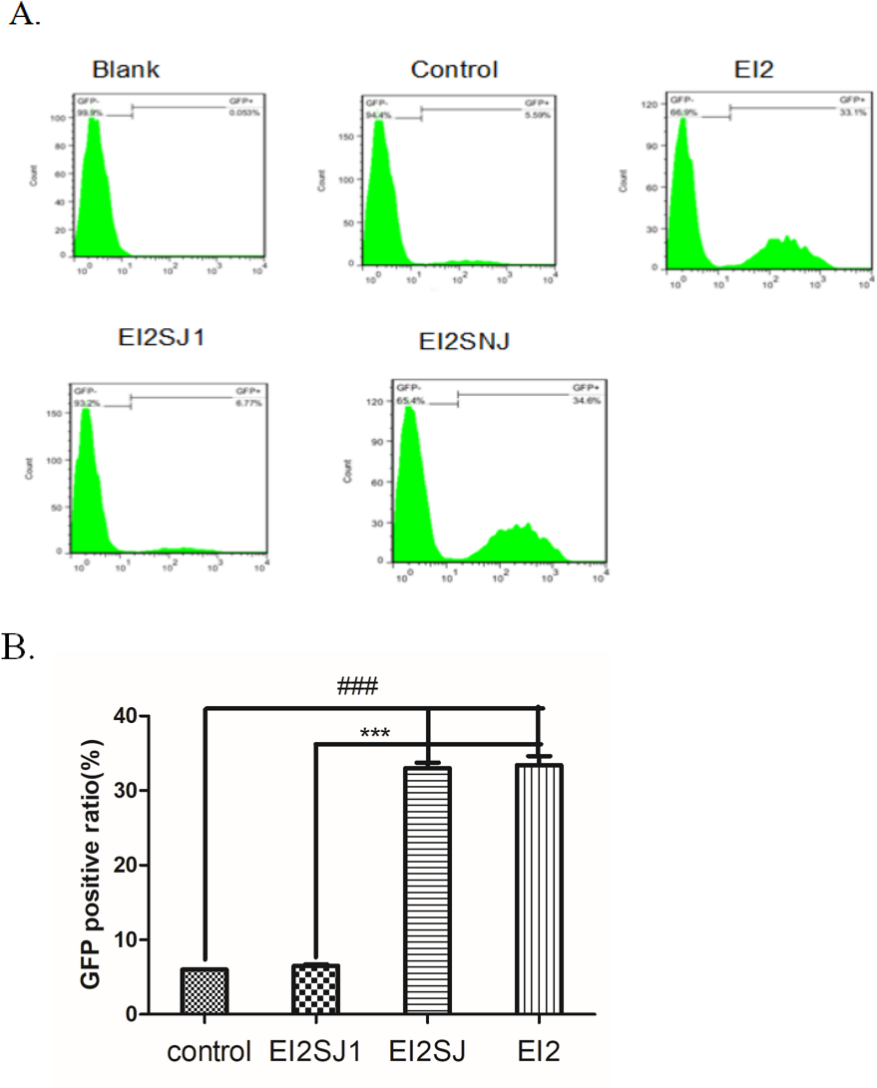
**(A)** GFP-JNK transfecting cells were treated with EI for 12hr. then analysed by flow cytometry. **(B)** GFP positive cell ratio for all groups. all data were derived form results of three independent experiments and are shown as mean ± SD. ^*^P<0.05, ^#^*P*<0.05, ^***^P<0.001, ^###^*P*<0.001 vs related group control or group EI2SJ1, respectively. We analyzed by one-way ANOVA followed by Tukey multiple comparison tests. Normal group (group N), Intralipid group (group F), EI of 9.52mM (group EI2), siRNA-JNK transfection plus EI of 9.80mM (group EI2SJ) and negative-siRNA-JNK plus EI of 9.52mM (group EI2NJ).

We transfected FNSCs with GFP-JNK to further determine whether EI induced cell apoptosis. The analysis of the GFP fluorescence images showed that EI increased the number of punctate GFP-JNK dots in a time-dependent manner (not shown). GFP-JNK transfecting cells were treated with EI for 12hr, the positive ratio of GFP-JNK was clearly decreased in EI2SJ1 group compared with EI2 group (Figure 5A, 5B). The result revealed that JNK was efficiently activated in group EI2NJ (negative-siRNA+EI2) compared with control group analyzed by flow cytometry (Figure 5B).

### Effects of JNK siRNA silencing on JNK protein expression and caspase3 activation levels

**Figure 6:**
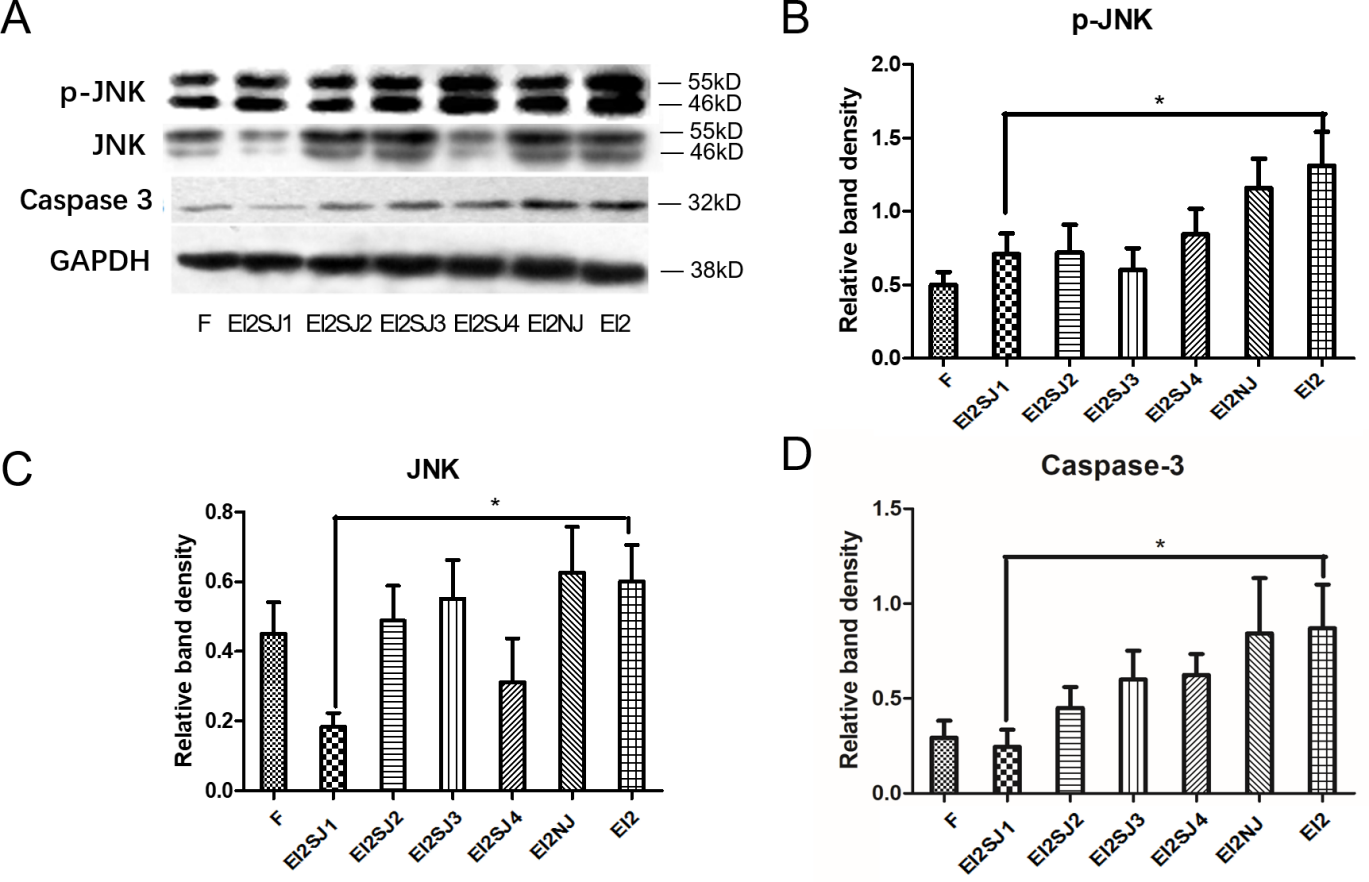
FNSCs transfection with siRNA-JNK (siRNA-JNK1, siRNA-JNK2, siRNA-JNK3, siRNA-JNK4) **(A)** Silent gene effect was validated by western blotting, and we choseeffectivesiRNA-JNK 1 gene (EI2S1) to do next experiment. **(B, C)** Protein expression of p-JNK and JNK in group N, group F, group EI2, group EI2SJ1 and group EI2NJ (negative-siRNA+EI2) were evaluated by western blotting. **(D)** The protein expression of Caspase3 in group N, group F, group EI2, group EI2SJ1 and group EI2NJ (negative-siRNA+EI2) were evaluated by western blotting. All data were derived form results of three independent experiments and are shown as mean ± SD. ^*^P<0.05 vs related group control, respectively. we analyzed by one-way ANOVA followed by Tukey multiple comparison tests. Normal group (group N), Intralipid group (group F), EI of 9.52mM (group EI2), siRNA-JNK transfection plus EI of 9.80mM (group EI2SJ) and negative-siRNA-JNK plus EI of 9.52mM (group EI2NJ).

Silent gene effect was validated by western blotting in protein of caspase3, p-JNK and JNK (Figure 6A). In order to corroborate the results from the pharmacological inhibition of JNK pathway, specific inhibition was performed by siRNA-targeting of JNK in FNSCs. Following 24 h of transfection with a specific siRNA against JNK, western blot analysis revealed that p-JNK and JNK was efficiently silenced in siRNA-JNK1 group (EI2SJ1), which was accompanied by a dose-dependent decrease in the protein levels of p-JNK and JNK compared with EI group (Figure 6B, 6C). Meanwhile, Caspase3 expression was efficiently decreased in siRNA-JNK1 group (EI2SJ1) in comparison with EI group (Figure 6D).

## DISCUSSION

Emulsified isoflurane (EI) is an emulsion formulation of isoflurane with both features of intravenous and inhalation anesthesia, the infused fat emulsion increases the isoflurane blood/gas partition coefficient [22]. Inhaled anesthetics could be safely injected into animals to produce anesthesia through emulsifying in fat [23]. previous studies indicated that EI had a shortened onset time compared with inhaled isoflurane [23] and a similar short onset time but a quicker recovery in comparison to propofol [24]. The successful application of EI in animals was for organ protection [25].

The difference of anesthetic partial pressure between alveolar gas and arterial blood has been shown to be a positive value during maintaining or deepening anesthesia by inhaled anesthetic [26] and might be a negative value when emulsified anesthetics are infused intravenously. The arterial partial pressures (indicating the partial pressure in the central nervous system after equilibration) of isoflurane between the intravenous approach and inhalation should be the same, because isoflurane dissolved in Intralipid make anesthesia through the same mechanism as isoflurane. Previous study showed that anesthetic micelles may link with lipid rafts to translate the signal to subcellular targets (e.g. mitochondria) to promote protection [27]. We all know that isoflurane is used in high doses or for long duration, especially if developing neuron is exposed to isoflurane, which can induce neuronal apoptosis [28].

In this study, our data revealed that EI induced FNSCs death by activating the ER stress response and sequent JNK activation. By *in vitro* approaches, we demonstrated that JNK blocker triggered the protective effect, which at least in part diminished the neurotoxicity of EI. Furthermore, our results indicated that EI-induced apoptosis activity may be activated by JNK pathway. JNKs are a family of protein kinases that play a central role in stress signaling pathways implicated in gene expression, neuronal plasticity, regeneration, cell death, and regulation of cellular senescence. There is a JNK pathway activation after exposure to different stressing factors, including cytokines, growth factors, oxidative stress, unfolded protein response signals or Aβ peptides [29].

Previous study demonstrated that isoflurane exposed to developing neurons, it can induce cell apoptosis [28]. Our study showed that EI activates JNK signaling and induces apoptosis in FNSCs for long-time durations, resulting in the inhibition of neurogenesis. Our findings raise the possibility that JNK inhibition has therapeutic potential in protecting FNSCs from the adverse effects of anesthetics.

A large number of *in vitro* and *in vivo* experiments have proven that isoflurane has a certain protective effect on many bio-stresses meanwhile producing neurotoxicity [30–32]. In our study, only concentration of or greater than 2.8mM would cause differences in proliferation inhibition rates of FNSCs compared with group N and group F. We studied the toxicity of FNSCs induced by high concentration of EI exposure, and provided theoretical support for further study on future clinical use of volatile anesthetics on human.

The ratio of Bax/Bcl-2 significantly increased in EI-treated cells, suggesting that EI modulates mitochondrial function to mediate FNSCs apoptosis. Meanwhile, mitochondria play a critical role in the regulation of various apoptotic processes, including drug-induced apoptosis [33]. Bcl-2 family members, such as pro-apoptotic protein Bax and anti-apoptotic protein Bcl-2 are critical regulators of the mitochondrial pathway [34], and Bax/Bcl-2 ratio reflected the apoptotic state of the FNSCs. These processes led to the activation of the executioner caspases-3, and upregulation of PARP and XBP1. We found that FNSCs treated with EI showed higher ER stress state than the control group. These data indicated that the mitochondrial pathway was involved in EI-induced apoptosis.

We detected a significant increase in IRE1 and XBP1 protein when FNSCs were treated with EI as compared with the control group. Inositol-Requiring Enzyme 1 (IRE1) is a key unfolded protein response (UPR) regulator [35]. IRE1 encodes an ER-resident transmembrane protein with an ER-luminal domain coupled to cytosolic kinase and endoribonuclease (RNase) domains [36–38]. ER stress-mediated JNK activation has also been reported to be associated with the activation of the transmembrane ER protein IRE1 [39]. IRE1 can initiate cell death in response to ER stress [41]. After activation by ER stress, IRE1 binds to tumor necrosis factor-receptor-associated factor 2 (TRAF2), and then recruits and activates apoptosis signal-regulating kinase 1 (ASK1), a mitogen-activated protein (MAP) kinase kinase kinase (MAP3K), this IRE1-TRAF2-ASK1 complex promotes activation of p38 and JNK during ER stress [40, 41–45]. ER stress-mediated apoptosis is triggered by the activation of ER membrane resident caspase-12 (mice) and caspase-4 (humans) and induction of Chop [46–48]. Once ER stress is activated, JNK phosphorylates the anti-apoptotic protein bcl-2. ER stress triggers autophagy through PERK/eIF2α, IRE1/JNK or potentially cleavage of eIF2α by caspase-3 [49]. In various physiological and pathological conditions, ER stress is a part of cell survival response, if the ER stress continues to expand, the ER function is unable to recover, and then the Caspase-dependent cell death follows.

Previous study showed that JNK pathway plays an important role in stimulating axonal growth and neuronal polarity [50]. EI could increase the p-JNK and JNK level. JNK inhibition by the specific inhibitor SP600125 rescued FNSCs from a decrease in survival and upregulated cell viability, so we speculated that JNK inhibition has therapeutic potential in protecting FNSCs from the adverse effects of drugs. so we need to prevent or ameliorate the adverse effects associated with exposure to such agents. JNK deficiency could result in death and severe nervous system defects in mouse embryos. The expressions of JNK protein in EI groups were higher than the controls. In order to further clarify whether JNK pathway was involved in EI-induced apoptosis, we preconditioned the cells with SP600125, a chemical blocker of JNK pathway, then the EI-induced proliferation and apoptosis were significantly decreased. It suggested that JNK signaling pathway was involved and might be used as a potential drug target for the treatment of neurotoxicity caused by anesthetics.

In conclusion, the present study demonstrated that specific inhibition of JNK activation by SP600125 treatment can effectively rescue FNSCs from apoptosis. Our study highlights the concept that suppressive JNK signaling pathway actually inhibits neural cell apoptosis, moreover, we used gene silencing technology to test the inhibition rates of cell proliferation with JNK siRNA silencing, which could be considered as further supporting evidence. Importantly, a study *in vivo* should be considered for future investigation to verify the findings *in vitro*. Nevertheless, the work reported here provides further understanding of FNSCs apoptosis modulated by EI with JNK signaling pathway involved. and provides critical preclinical evidence showing that specific inhibition of JNK activation by SP600125 treatment may be a novel promising disease-modifying therapeutic strategy for the treatment of developmental nerve damage.

## MATERIALS AND METHODS

### FNSCs cultures

Primary embryonic neural stem cells were isolated from fetal brain cortex of Sprague-Dawley rats on day 14 of gestation (Invitrogen, USA). Isolated FNSCs were propagated on CELL start™ CTS™-coated T25 flask to maintain an adherent culture. The cells were cultured in KnockOut™ DMEM/F12 medium buffered with 2% StemPro® Neural Supplement, 2mM L-glutamine, 20ng ml^-1^ bFGF and 20ng ml^-1^ EGF, incubated at 37°C in a humidified atmosphere containing 5% CO_2_. The culture medium was replaced every 2∼3 days. After culture and passage, the rat FNSCs can propagate to 3∼5 passages without differentiation with more than 75% of cells retaining their undiffenentiation phenotype. The cells were seeded in 96-well plates and incubated 24h before treated with EI. Reagents were from the following sources: Intralipid from Sino-Swed Pharmaceutical Corp. Ltd; EI from West China hospital; CELL start™ CST™, StemPro Accutase Cell Dissociation Reagent, L-glutamate, KnockOut™ DMEM/F-12, EGF Recombinant Human Protein, FGF-Basic (AA 10-155) Recombinant Human Protein, Lipofectamine 2000 Transfection Reagent from Invitorgen; SP600125 from Sigma Aldrich; Annexin V-FITC/PI from BD; Caspase 3 and JNK1+JNK2 primary antibody from Abcam; siRNA-JNK from Genepharma.

### Different concentrations of EI treating and JNK pathway

Cells cultured in same batch were randomly divided into 6 groups (n=8): normal group (group N), Intralipid group (group F), EI of 7.56mM (group EI1), EI of 9.52mM (group EI2), EI of 11.48mM (group EI3) and EI of 9.52mM plus SP600125 of 50 μM (group EI2SP). SP600125 is a potent inhibitor of JNKs that used widely in biochemical studies. All groups were incubated for 12h, and then the cell viability, cell apoptotic rates, the expressions of JNKs and apoptosis-related protein Caspase3 were tested.

### EI treating with JNK blocker

The cultured rat FNSCs were randomly divided into 4 groups (n=8): normal group (group N), Intralipid group (group F), EI of 9.52mM (group EI2) and EI of 9.52mM plus JNK blocker SP600125 of 50 μM (group EI2SP). The cell viability, cell apoptotic rates and the expression of apoptosis-related protein Caspase3, IRE1, PARP, Bax, Bcl-2 and XBP1 were observed respectively.

### EI treating under JNK gene silencing and measurement of apoptosis-related protein

Small interfering (si) RNA was used to knock down JNK expression. Cells cultured in same batch were randomly divided into 5 groups (n=8): normal group (group N), Intralipid group (group F), EI of 9.52mM (group EI2), siRNA-JNK transfection plus EI of 9.52mM (group EI2SJ) and negative-siRNA-JNK plus EI of 9.52mM (group EI2NJ), then the inhibition rates of cell proliferation were tested. The JNK and control siRNAs were purchased from Cell Signaling Technology (Beverly, MA, USA). and control (sequence: CCUACGCCAAUUUCGUdTdT) siRNAs were synthesized by Bioneer. The transfection experiments were performed using Lipofectamine 2000 reagent according to the manufacturer’s instructions (Invitrogen, USA).

Effect of JNK1/2 silencing (via siRNA) on FNSCs cellular activity. Following siRNA transfection (24 h), western blotting revealed that the siRNA-JNK inhibited JNK total protein expression compared with control transfection experiments.

## MOLECULAR BIOLOGY TECHNOLOGY

### MTT assay

The cellular effects of EI and cell viability were evaluated by 3-(4, 5-dimethyithiazol-2-yl)-2,5-diphenyl-tetra-zolium bromide (MTT) reduction assay. FNSCs were seeded in 96-well plates and allowed to grow for 24h before treatments. Thereafter, 100 μl working solution obtain different concentrations of EI was added into the well. Treatment duration was 12 h. After each cell group establishment, a 10% MTT (Sigma Aldrich, USA) solution (5 mg/mL) diluted in PBS was added to each well. The cultures were immediately incubated at 37°C and protected from light for another 12 hours. After incubation, the media was removed and refilled dimethyl sulfoxide (Sigma) of 150 μl and then placed on shaker for 10 min. And, the optical density was measured at wavelength of 450 nm using absorbance readers.

### Detection of cell apoptosis by annexin V staining

FNSCs cultured in 24-well plates were treated with EI or Intralipid. Cells undergoing apoptosis at the indicated time were identified using FITC Annexin V Apoptosis Detection Kit (BD), following the manufacturer's instructions.

### Western blot

The expressions of all proteins were observed by western blot. Cells were washed in phosphate buffered saline, and resuspended at room temperature. After treatment on ice for 30 min, the lysate was centrifuged at 14,000 x g at 4°C. Protein concentration was measured with the Bradford protein assay reagent and bovine serum albumin as a standard. The membranes were incubated overnight at 4°C with the designated primary antibodies and secondary antibodies at room temperature for 2 h. GAPDH was used as the control.

### Statistics analysis

Data were presented as means±S.D. All statistical tests were performed in Graphpad Prism 5.0. m. Measurement data were compared by one-way analysis of variance (ANOVA) followed by Tukey multiple comparison tests. Statistical significance was set at a level of *P* < 0.05.
